# Efficiently Hiding Sensitive Itemsets with Transaction Deletion Based on Genetic Algorithms

**DOI:** 10.1155/2014/398269

**Published:** 2014-09-01

**Authors:** Chun-Wei Lin, Binbin Zhang, Kuo-Tung Yang, Tzung-Pei Hong

**Affiliations:** ^1^Innovative Information Industry Research Center (IIIRC), School of Computer Science and Technology, Harbin Institute of Technology Shenzhen Graduate School, Shenzhen 518055, China; ^2^Shenzhen Key Laboratory of Internet Information Collaboration, School of Computer Science and Technology, Harbin Institute of Technology Shenzhen Graduate School, Shenzhen 518055, China; ^3^Medical School, Shenzhen University, Shenzhen 518060, China; ^4^Department of Computer Science and Information Engineering, National University of Kaohsiung, Kaohsiung 811, Taiwan; ^5^Department of Computer Science and Engineering, National Sun Yat-sen University, Kaohsiung 804, Taiwan

## Abstract

Data mining is used to mine meaningful and useful information or knowledge from a very large database. Some secure or private information can be discovered by data mining techniques, thus resulting in an inherent risk of threats to privacy. Privacy-preserving data mining (PPDM) has thus arisen in recent years to sanitize the original database for hiding sensitive information, which can be concerned as an NP-hard problem in sanitization process. In this paper, a compact prelarge GA-based (cpGA2DT) algorithm to delete transactions for hiding sensitive itemsets is thus proposed. It solves the limitations of the evolutionary process by adopting both the compact GA-based (cGA) mechanism and the prelarge concept. A flexible fitness function with three adjustable weights is thus designed to find the appropriate transactions to be deleted in order to hide sensitive itemsets with minimal side effects of hiding failure, missing cost, and artificial cost. Experiments are conducted to show the performance of the proposed cpGA2DT algorithm compared to the simple GA-based (sGA2DT) algorithm and the greedy approach in terms of execution time and three side effects.

## 1. Introduction

With the rapid growth of data mining technologies in recent years, useful and meaningful information can thus be easily discovered for the purpose of decision making in different domains. The discovered information can be mostly classified into association rules [1–5], sequential patterns [6–9], classification [10–12], clustering [13, 14], and utility mining [15–18], among others. Among them, mining association rules method is the most common way to find the potential relationships between the purchased items or goods in a very large database. Some applications require protection against the disclosure of private, confidential, or secure data. For example, social security numbers, address information, credit card numbers, and purchasing behaviors of customers can be considered as the confidential, private, or privacy information.

Instead of personal information, privacy issue can be extended to business. Based on business purposes, shared information among companies may be extracted and analyzed by other partners, thus causing the security threats. Privacy-preserving data mining (PPDM) [19–22] was proposed to reduce privacy threats by hiding sensitive information while allowing required information to be discovered from databases. Such data may implicitly contain confidential information that will lead to privacy threats if it is misused. Heuristic methods [20, 21, 23–26] have been proposed to choose the appropriate data for sanitization in order to hide the sensitive information. During the procedure to hide the sensitive information, side effects of missing cost and artificial cost are thus generated and should be concerned in PPDM. The optimal way to select the sensitive information to be hidden is, however, concerned as the NP-hard problem in sanitization process [22, 27]. Genetic algorithms (GAs) [28] are able to find optimal solutions using the principles of natural evolution. The amount of chromosomes is thus required to process the several operations in evaluation process of simple GAs.

To solve the limitations of traditional GA-based algorithms with high requirements of memory and computations at each evolutionary process, the compact GA (cGA) mechanism [29] and the prelarge concept [30] are adopted in the proposed cpGA2DT algorithm. Based on the cGA mechanism, only two chromosomes are competed to each other at each iteration. The probabilities of transactions to be selected are increased along with the winner chromosome. The probabilities of transactions to be selected are, however, decreased along with the loser chromosome. Since only two chromosomes are generated for the competition, the memory requirements of populations can be greatly reduced. In addition, a flexible fitness function is designed to evaluate three side effects at each evolutionary process. This procedure causes the computations of multiple database rescans. The prelarge concept is adopted in the proposed cpGA2DT algorithm to find the prelarge itemsets [30, 31] in advance, thus reducing the computations of multiple database rescans at each evolution. To the best of our knowledge, this is the first approach to solve the limitations by considering both the time and the space complexities with transaction deletion for hiding sensitive itemsets. A straightforward approach (greedy) and a simple GA-based algorithm are also designed as a benchmark to evaluate the performance of the proposed cpGA2DT in regard to the execution time and the number of three side effects in the experiments. Contributions of this paper can be illustrated as follows.

1. Most past approaches applied heuristic ways to sanitize the original database for the purpose of hiding sensitive itemsets by deleting partial items. In this paper, a GA-based approach is thus proposed to optimize the selected transactions to be deleted, thus minimizing the side effects in PPDM.
2. It requires the amount of memory in evaluation process based on traditional GA approach. In this proposed approach, cGA is applied to reduce the population size based on probability distribution to select the appropriate transactions to be deleted.
3. The prelarge concept is used in the proposed algorithm to reduce the execution time for database rescan in chromosome evaluation.
4. An evaluation function with three adjustable weights is designed in the evaluation process to minimize the side effects of PPDM.

The remainder parts of this paper are organized as follows. Related works are described in Section 2; preliminary of PPDM is mentioned in Section 3. The proposed approach is illustrated in Section 4. An example is given in Section 5. Experiments are conducted in Section 6. Conclusion is given in Section 7.

## 2. Review of Related Works

Related works of genetic algorithms, data sanitization, and prelarge concept are briefly reviewed in this section.

### 2.1. Genetic Algorithms

**Figure 1:**
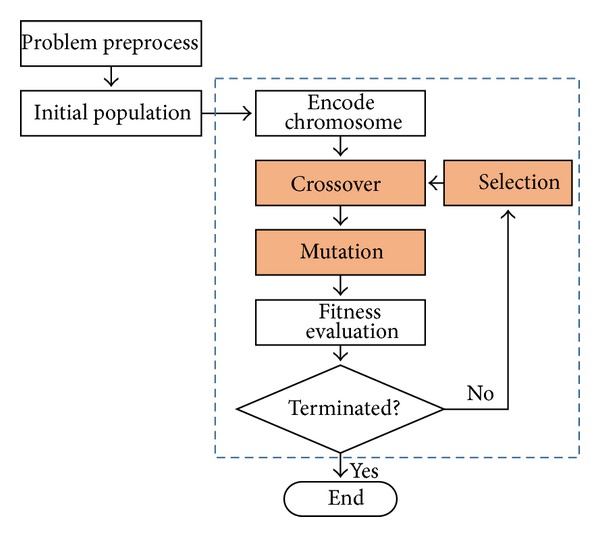
Flowchart of GAs.

Holland applied the natural selection and the survival of the fittest of Darwin theory and proposed the evolutionary computation of genetic algorithms (GAs) [28]. GAs are the search techniques, which are designed and developed to find a set of feasible solutions in a limited amount of time [32, 33]. According to the principle of survival of the fittest, GAs generate the next population by various operations with each individual in the population representing a set of possible solutions. Three basic operations including crossover, mutation, and selection are performed on chromosomes for the next generations. Each chromosomea is then evaluated by the designed fitness function. This procedure is recursively processed until the predefined termination criteria are achieved. Flowchart of GAs is shown in Figure 1.

Traditional GAs have to generate the size of populations for the purpose of performing crossover, mutation, and selection operations for the next generations, thus causing memory lack problem. Compact genetic algorithm (cGA) was thus proposed to simulate traditional GAs with only the probability vector for selection operation and population size without the crossover and mutation operations in order to generate two individuals (or chromosomes) at competition [29]. The probability of the* i*th vector in the winner chromosome is increased, but the loser probability is decreased. A cGA algorithm can reduce the memory requirements without the crossover and mutation operations but still can approximately mimic the behaviors of traditional GAs.

### 2.2. Data Sanitization

Data mining [1, 34–37] is progressively developed to extract useful and meaningful information or rules from a very large database. The misuse of data mining techniques may, however, lead to security threats and privacy concerns. Privacy-preserving data mining (PPDM) [19, 23, 24, 38] was thus proposed to hide the confidential, private, or secure information before it is published in public or shared among alliances. Most approaches were proposed to perturb the original database for the purpose of hiding sensitive information in PPDM. Agrawal and Srikant introduced a quantitative measure to evaluate the utility of PPDM methods [19]. Lindell and Pinkas stated hiding confidential information on the union of shared databases among two parties without revealing any unnecessary information [20]. Oliveira and Zaïane, respectively, designed the multiple-rule hiding MinFIA, MaxFIA, and IGA algorithms to efficiently hide sensitive itemsets and introduced the performance measures for three side effects [39]. Dasseni et al. then proposed a hiding approach based on the hamming-distance approach to decrease the confidence or support values of association rules for hiding sensitive information [40]. Three heuristic algorithms are designed, respectively, to increase the supports of antecedent parts, to decrease the supports of consequent parts, and to decrease the support of either the antecedent or the consequent parts until the supports or confidences of association rules below the threshold values. Amiri then proposed aggregate, disaggregate, and hybrid approaches to hide multiple sensitive rules [23]. The designed aggregate approach computes the union of the supporting transactions for all sensitive itemsets. The transactions with the most sensitive and the least sensitive itemsets are thus removed to hide the sensitive information. The disaggregate approach aims to remove individual items from transactions and then remove whole transactions, thus reducing side effects of PPDM. Hybrid one is to combine the previous designed algorithms to firstly identify sensitive transactions and secondly to delete items from those of transactions until the sensitive information has been hidden. Many heuristic approaches are still being developed in progress for the purpose of hiding different types of knowledge in PPDM [21, 26, 41].

The optimal sanitization of databases is regarded to be an NP-hard problem [22, 27]. Genetic algorithms (GAs) were usually used to find optimal solutions in the least amount of time [28]. Fewer studies have adopted GAs to find optimal solutions to hide sensitive information. Han and Ng proposed secure protocols for rule discovery based on private arbitrarily partitioned data among two parties without compromising their data privacy using GAs [42]. It uses the true positive rate multiplied by the true negative rate to define the fitness function for evaluating the goodness of each decision rule. Dehkordi et al. designed three multiobjective methods to partially remove the items from the original database [43]. Only the number of modified transactions is considered in the fitness function for evaluation. The other side effects of missing cost and artificial cost thus arose in the evaluation process. In this paper, three side effects are concerned in the designed fitness function for hiding sensitive itemsets with transaction deletion based on cGA algorithm.

### 2.3. Prelarge Concept

**Figure 2:**
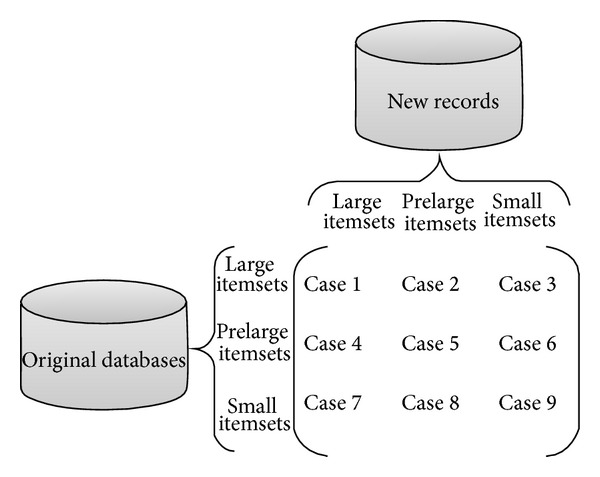
Nine cases arise as a result of transaction deletion.

Data mining techniques are used to discover useful and meaningful information or rules to aid managers in making efficient decisions in many different domains. Most data mining techniques handle, however, the static database to extract the required information. Cheung et al., respectively, designed FUP [44] and FUP2 [45] concepts to maintain and update the discovered information in dynamic databases. The original database is still, however, required to be rescanned based on the FUP and FUP2 concepts in the updating process. Hong et al. proposed prelarge concepts [30, 31] for the purpose of efficiently updating the discovered information without rescanning the original database each time. Prelarge itemset is not large itemset but has high potential to be large in the future through the data insertion or deletion process. Upper (the same as the minimum support threshold in conventional mining algorithms) and lower support thresholds are used to define the large and prelarge itemsets. Prelarge itemsets are used as a buffer to reduce the movement of an itemset directly from large to small and vice versa. For transaction deletion based on prelarge concept [30], nine cases thus arose and are shown in Figure 2.

From Figure 2, cases 2, 3, 4, 7, and 8 do not affect the final frequent itemsets of association rules. Case 1 may remove some discovered frequent itemsets of association rules. Cases 5, 6, and 9 may produce new frequent itemsets of association rules. If all frequent or prelarge itemsets are prestored from the original database, cases 1, 5, and 6 can be easily maintained and updated. An itemset in Case 9 cannot possibly be a large itemset in the updated database as long as the number of deleted transactions is a considerably small proportion of the original databases, which can be defined as [30]

(1) $$\begin{array}{c} f\leq \frac{\left(S_{u}-S_{l}\right)\times \left|D\right|}{S_{u}}, \end{array}$$

where *S*_*l*_ is a lower support threshold, *S*_*u*_ is an upper support threshold, and |*D*| is the number of transactions in databases. If the number of deleted transactions satisfies the above condition, which is smaller than the safety bound *f*, an itemset in Case 9 is absolutely not large in the updated databases. It is thus unnecessary to rescan the original databases. In the proposed cpGA2DT, the prelarge concepts are adopted to reduce the database rescan in the evaluation process, thus speeding up computations.

## 3. Preliminaries

Before sanitization process to hide the sensitive itemsets, frequent itemsets can be discovered by data mining techniques. Let *I* ∈ {*i*_1_, *i*_2_,…, *i*_*n*_} be the set of items in the database *D*; a database *D* consists of several transactions as *D* ∈ {*t*_1_, *t*_2_,…, *t*_*m*_}, in which each transaction is a set of items. A minimum support threshold is set at *σ*. Denote a support of an item (itemset) by sup⁡(*i*_*j*_). An item (itemset) is denoted by freq(*i*_*j*_) if it is considered as a large or frequent item (itemset) as freq(*i*_*j*_) = sup⁡(*i*_*j*_)/|*D*| ≥ *σ*.

**Figure 3:**
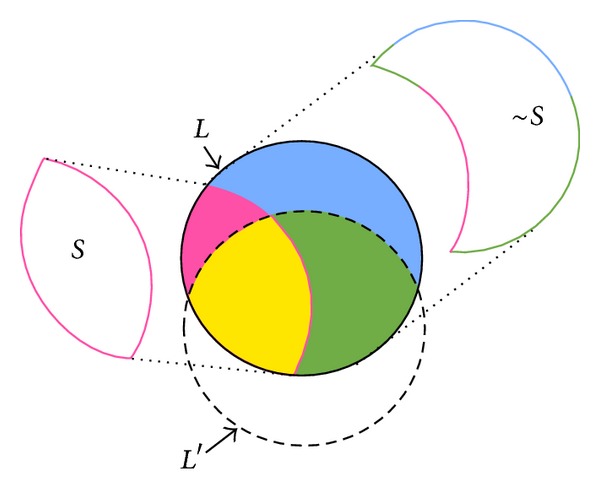
The relationship of itemsets before and after the PPDM process.

In PPDM, it is required not only to hide sensitive itemsets but also to minimize the side effects. The relationship of itemsets before and after the PPDM process can be seen in Figure 3, where *L* represents the large itemsets of *D*, *S* represents the sensitive itemsets defined by users that are large, ~*S* represents the nonsensitive itemsets that are large, and *L*′ is the large itemsets after some transactions are deleted.

**Figure 4:**
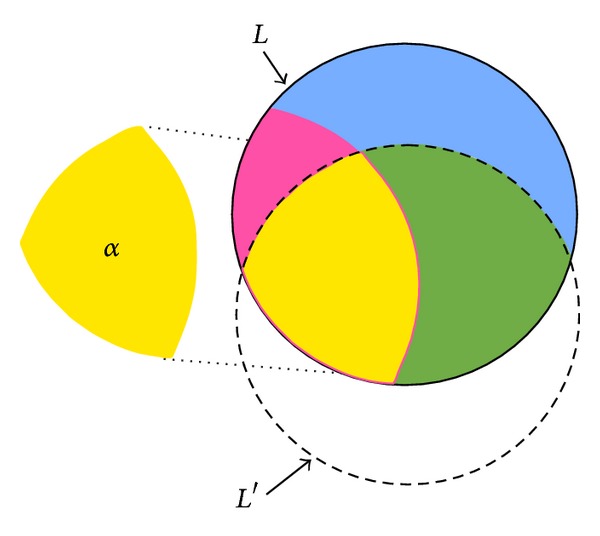
The set of sensitive itemsets that fail to be hidden.

Let *α* be the number of sensitive itemsets that fail to be hidden. Thus, the number of sensitive itemsets should ideally be zero after the database is sanitized. The set of sensitive itemsets is shown in Figure 4, in which *α* part is the interaction of *S* and *L*′.

**Definition 1 .**

The hiding failure of the sensitive itemsets in PPDM is defined as *α*, in which *α* = *S*∩*L*′.

**Figure 5:**
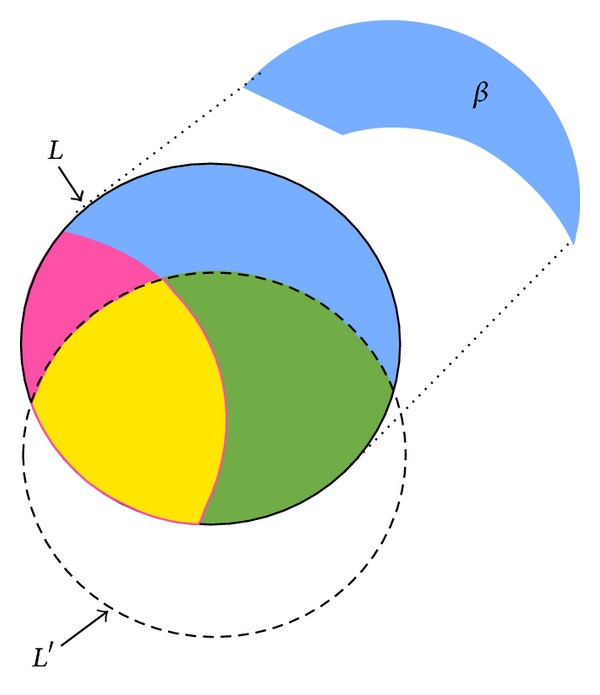
The set of sensitive itemsets that fail to be hidden.

Another evaluation criterion is the number of missing itemsets, which is denoted by *β*. A missing itemset is a nonsensitive large itemset in the original database but is not extracted from the sanitized database. This side effect is shown in Figure 5, in which the *β* part is the difference of ~*S* and *L*′.

**Definition 2 .**

The missing itemsets in PPDM are defined by *β*, in which *β* = ~*S* − *L*′ = (*L* − *S*) − *L*′.

**Figure 6:**
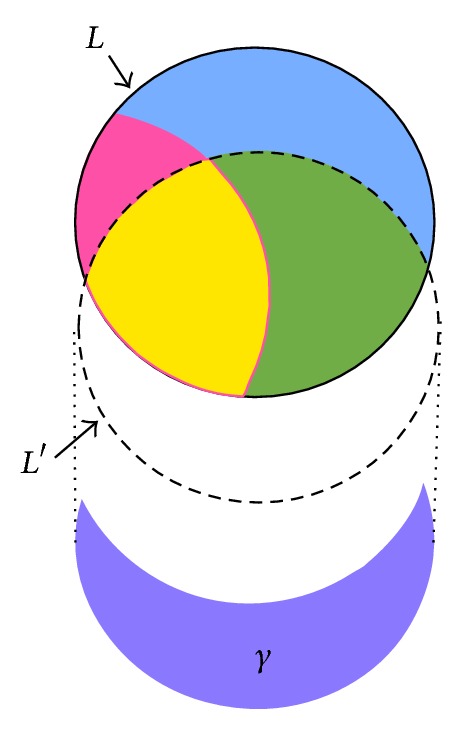
The set of artificial itemsets.

The last evaluation criterion is the number of artificial itemsets, which is denoted by *γ*. It represents the set of large itemsets appearing in the sanitized database but not belonging to the large itemset in the original database. This side effect is shown in Figure 6, in which the *γ* part is the difference of *L*′ and *L*.

**Definition 3 .**

The artificial itemsets in PPDM are defined as *γ*, in which *γ* = *L*′ − *L*.

Hiding sensitive itemsets or information is not only one purpose of PPDM but also minimizing the above side effects for data sanitization.

## 4. Proposed Compact Prelarge Genetic Algorithm to Delete Transactions (cpGA2DT)

In this paper, a cpGA2DT approach is thus proposed to find the appropriate transactions to be deleted for hiding sensitive itemsets. The sensitive itemsets to be hidden can be defined below.

**Definition 4 .**

Suppose that a set of HS consist of the amounts of sensitive itemsets to be hidden; thus HS = {si_1_, si_2_,…, si_*k*_}.

In the proposed cpGA2DT for hiding the sensitive itemsets through transaction deletion, the support count of a sensitive itemset must be below the minimum support threshold, in which each transaction to be deleted must contain any of the sensitive itemsets in HS.

**Definition 5 .**

Suppose an original database *D* = {*T*_1_, *T*_2_,…, *T*_*n*_}; a database *D*′ is thus projected from *D*, in which each *T*_*j*_ in *D*′ must consist of any of the sensitive itemsets in HS.

In GAs, a chromosome corresponds to a possible solution. Suppose that *m* is appropriate transactions from *D*′ to be deleted for hiding the sensitive itemsets. A chromosome with *m* genes is thus designed. Each gene represents a possible transaction to be deleted as a positive integer of transaction ID (TID) value or* null*.

**Definition 6 .**

Suppose a projected database *D*′ = {*T*_1_, *T*_2_,…, *T*_*n*_}, in which each *T*_*j*_ represents a transaction ID. Suppose that *m* is appropriate transactions to be deleted; a chromosome *c*_*i*_ is a set of *m* gens. Each *m* in *c*_*i*_ is represented as a transaction *T*_*j*_ or* null*.

In GAs, a flexible fitness function with three adjustable weights to evaluate the goodness of chromosomes is thus designed.

**Definition 7 .**

A fitness function to evaluate the goodness of a chromosome *c*_*i*_ is defined as

(2) $$\begin{array}{c} \text{fitness}\left(c_{i}\right)=w_{1}\times \alpha +w_{2}\times \beta +w_{3}\times \gamma , \end{array}$$

where *w*_1_, *w*_2_, and *w*_3_ are the weighting parameters. The *α*, *β*, and *γ* are the hiding failure, missing cost, and artificial cost. Details of the notations and the proposed cpGA2DT algorithm are described in Algorithm 1.

### 4.1. Proposed cpGA2DT Algorithm

The designed cpGA2DT algorithm is described in Algorithm 1.

For the proposed cpGA2DT, it adopts both the compact GA and prelarge concepts to reduce not only the computations of database rescan but also the population size at each evaluation. Prelarge itemsets (PL) act like buffers and are used to reduce the movement of itemsets directly from large to small and vice versa when transactions are deleted (in steps (1) and (2)). In competition process, only two individuals are used for competition (in step (8)). This approach can reduce the population size to speed up the evaluation process. When the termination condition is not satisfied, two chromosomes are then generated again, respectively, to increase the probability of selected transactions in the winner chromosome but decrease the probability of selected transactions in the loser chromosome.

## 5. An Illustrated Example

In this section, an example is given to demonstrate the proposed cpGA2DT for privacy-preserving data mining. Assume that an original database contains 10 transactions shown in Table 1.

Also assume that the set of sensitive itemsets is defined as {*be*, *bce*} to be hidden. The minimum support threshold is set at 40%. The proposed algorithm is then processed as follows. The transactions with any of the sensitive itemsets in Table 1 are then projected. In this example, transactions 2, 3, 4, 5, 7, and 8 are then projected to form another projected database. The initial probabilities of those five transactions are initially set at 0.5. The lower support threshold for deriving the prelarge itemsets in this example is calculated as *S*_*l*_ = *S*_*u*_ × (1 − *m*/|*D*|)( = 0.4)×(1 − 4/10) ( = 0.24). The database is scanned to find the large and prelarge itemsets. The results are, respectively, shown in Tables 2 and 3.

Two chromosomes (individuals) are then generated randomly according to the probability vector with 4 genes. The results are then shown in Table 4.

The chromosomes in Table 4 are then competed by the designed fitness function. In this example, the weights for three factors are, respectively, set as 0.5, 0.3, and 0.2. Take *C*_*A*_ as an example to illustrate the evolutionary process. The number of hiding failures for *C*_*A*_ is 0 since all sensitive itemsets (*be*,* bce*) are completely hidden; the number of missing itemsets of *C*_*A*_ is 3 (itemsets* e*,* bc*, and* ce* are missing), and the number of artificial itemsets of *C*_*A*_ is 1 (itemset* ac* arose). The fitness value of *C*_*A*_ is calculated as fitness(*C*_*A*_) = 0.5 × 0 + 0.3 × 3 + 0.2 × 1 (=1.1). The *C*_*B*_ is processed in the same way, and fitness(*C*_*B*_) = 0.5 × 0 + 0.3 × 3 + 0.2 × 0 (=0.9). In the competition process, the *C*_*B*_ is better than *C*_*A*_; the probabilities of transactions 2, 3, 4, and 7 are then, respectively, increased and updated in the probability vector by 0.5 + 1/6 (=0.667); the probabilities of transactions 2, 5, 7, and 8 are then, respectively, decreased and updated in the probability vector by 0.5 – 1/6 (=0.33). After that, the probability vector is updated and shown in Table 5.

Steps (5) to (8) are then, recursively, processed until the termination condition is satisfied. In this example, three criteria are used as the termination conditions. The criteria are as follows. The fitness function value of the best chromosome is 0; or a predefined number of generations is achieved; or the probability vector is converged. After the evolutionary process, the top-4 transactions with high probabilities in the probability vector are then selected as the transactions to be deleted in the sanitization process.

## 6. Experimental Results

Experiments are conducted to show the performance of the proposed cpGA2DT, which was performed on a Pentium IV processor at 2 GHz and 512 M of RAM running on the Mandriva platform. A greedy approach and a simple GA-based algorithm [46] are also designed as a benchmark to be compared with the proposed algorithm. For the greedy approach, it scans the transactions from top to down to directly delete the transactions with sensitive itemsets. The termination of the greedy algorithm is the number of the deleted transactions, which is predefined by users. A simple GA-based approach uses simple GAs to hide the sensitive information. Three real databases mushroom [47], BMS-WebView1 [48], and BMS-WebView2 [48] are used to evaluate the performance of the proposed cpGA2DT in terms of the execution time and the number of three side effects. The weights for three side effects *α*, *β*, and *γ* are set at 0.5, 0.25, and 0.25, which can be adjusted by users. Details of the three databases used in the experiments are shown in Table 6.

### 6.1. Execution Time

**Figure 7:**
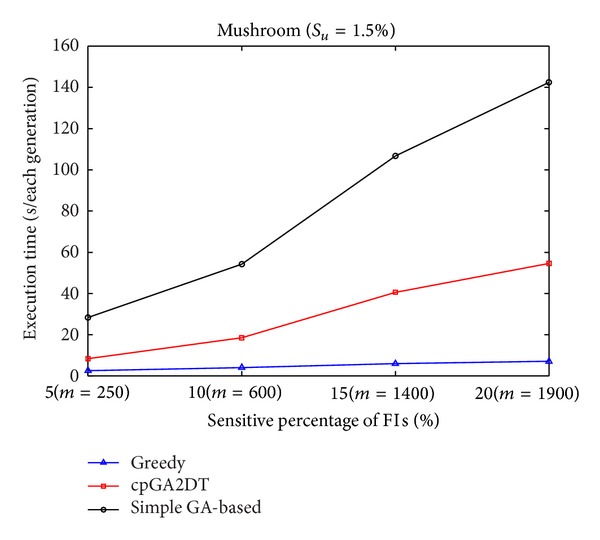
Comparisons of execution time at various sensitivity percentages for mushroom database.

**Figure 8:**
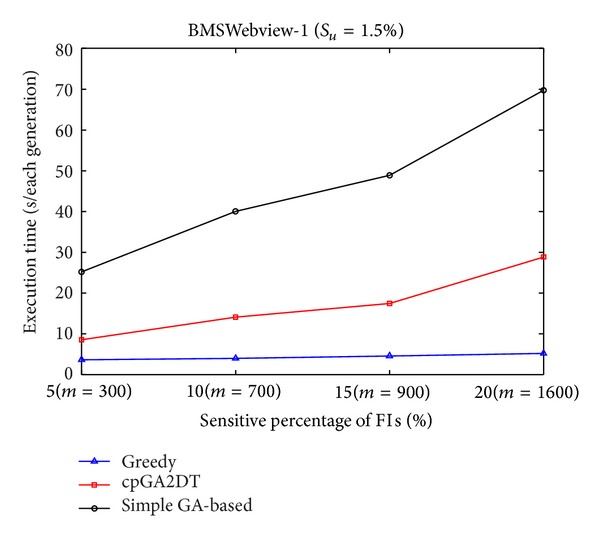
Comparisons of execution time at various sensitivity percentages for BMSWebview-1 database.

**Figure 9:**
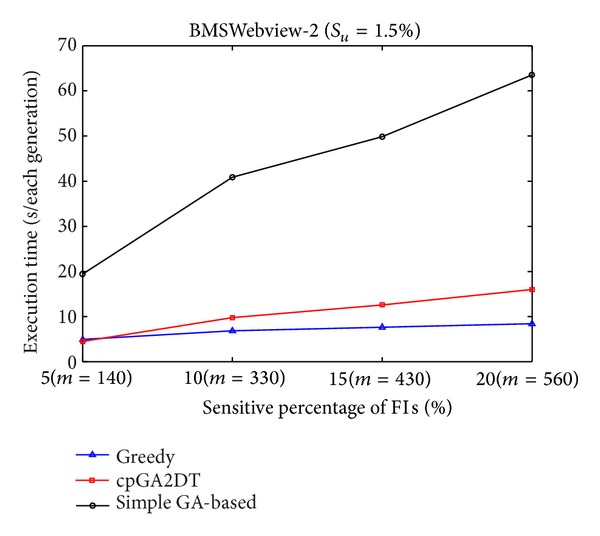
Comparisons of execution time at various sensitivity percentages for BMSWebview-2 database.

Execution times obtained the proposed cpGA2DT; greedy and simple GA-based algorithms are then compared at various sensitivity percentages of the sensitive itemsets for three databases. Results are shown in Figures 7, 8, and 9. The *S*_*u*_ is initially set at 1.5%. According to predefined number of transactions to be deleted (the size of chromosome) in the original database, the *S*_*l*_ is easily retrieved for deriving the prelarge itemsets, thus speeding up the execution time without computations of database rescan.

**Figure 10:**
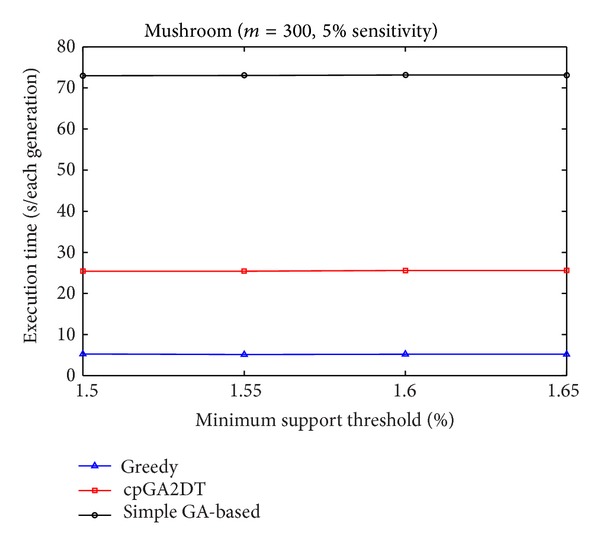
Comparisons of execution time at various minimum support thresholds for mushroom database.

**Figure 11:**
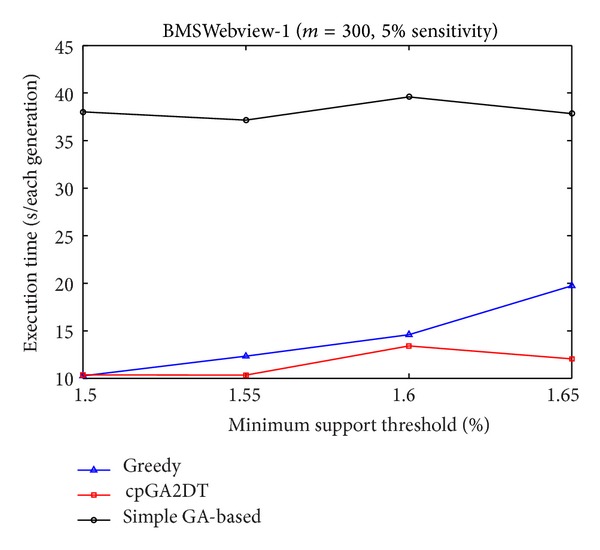
Comparisons of execution time at various minimum support thresholds for BMSWebview-1 database.

**Figure 12:**
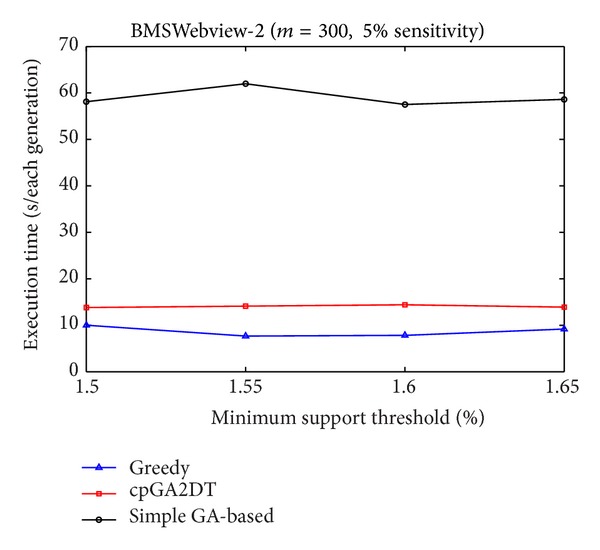
Comparisons of execution time at various minimum support thresholds for BMSWebview-2 database.

From Figures 7 to 9, it is obvious to see that the straightforward greedy approach has the best performance in execution time since it does not consider any side effects but directly delete the transactions for the purpose of hiding sensitive itemsets. The proposed cpGA2DT can greatly reduce the execution time compared to the simple GA-based algorithm since for cpGA2DT it is unnecessary to rescan the original database for evaluating fitness at each iteration. Experiments are then conducted to show the execution times for three algorithms at various minimum support thresholds. The results are then shown in Figures 10, 11, and 12.

Form Figures 10 and 12, it is obvious to see that the greedy approach has the best performance of execution time at various minimum support thresholds. The proposed cpGA2DT has the best performance in BMSWebview-1 database. The simple GA-based algorithm still has the worst performance in execution time since it requires to rescan the original database to evaluate the goodness of fitness at each iteration. The side effects of hiding failure, missing cost, and the artificial cost are also evaluated to show the performance of the proposed cpGA2DT. The descriptions are given as follows.

### 6.2. Hiding Failure (HF)

**Figure 13:**
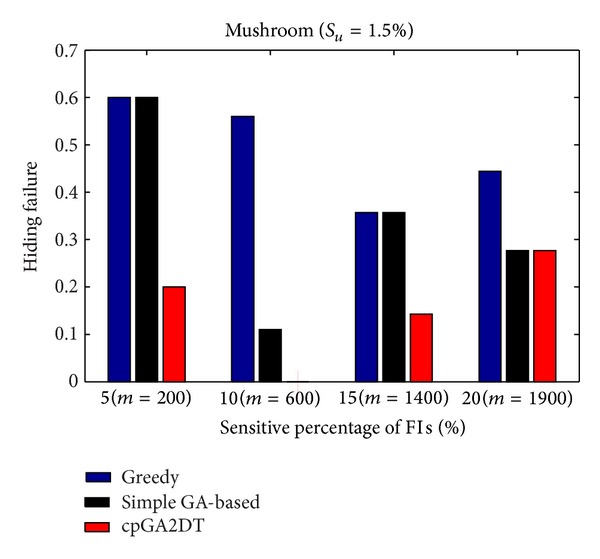
Comparisons of hiding failure at various sensitivity percentages of the frequent itemsets for mushroom database.

**Figure 14:**
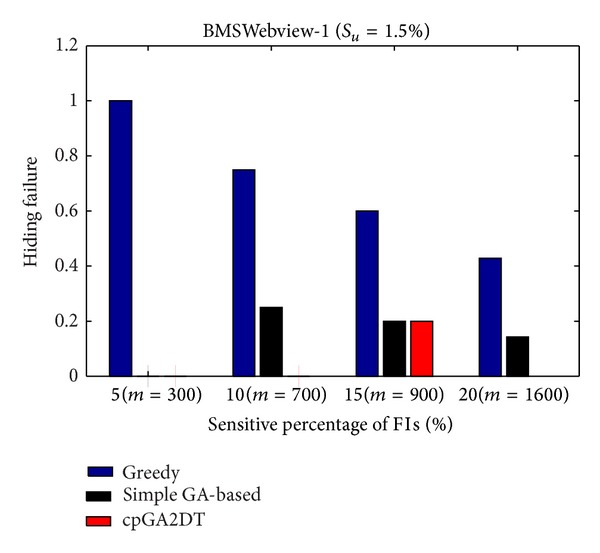
Comparisons of hiding failure at various sensitivity percentages of the frequent itemsets for BMSWebview-1 database.

**Figure 15:**
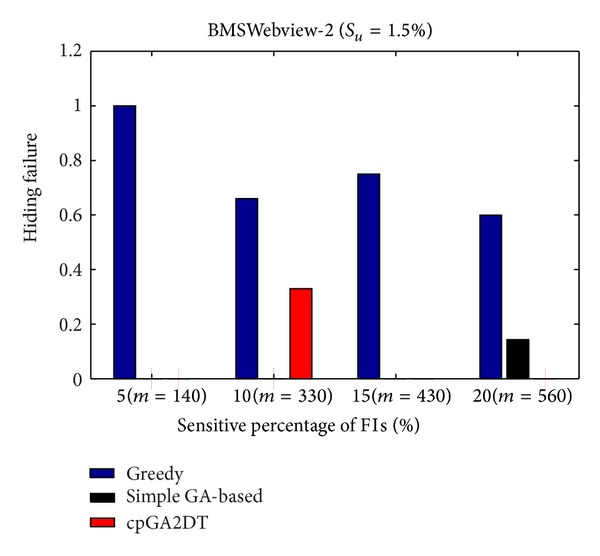
Comparisons of hiding failure at various sensitivity percentages of the frequent itemsets for BMSWebview-2 database.

The hiding failure is one of the side effects to evaluate whether the sensitive information has been successfully hidden before and after sanitization process, which can be calculated as

(3) $$\begin{array}{c} \text{HF}=\frac{\left|\text{HS}\left(D^{*}\right)\right|}{\left|\text{HS}\left(D\right)\right|}, \end{array}$$

where |HS(D*)| is the number of sensitive itemsets after sanitization process and the |HS(*D*)| is the number of sensitive itemsets before sanitization process. The hiding failure obtained three algorithms at various sensitivity percentages of the sensitive itemsets for three databases with *S*_*u*_ (= 1.5%). The results are then shown in Figures 13, 14, and 15.

**Figure 16:**
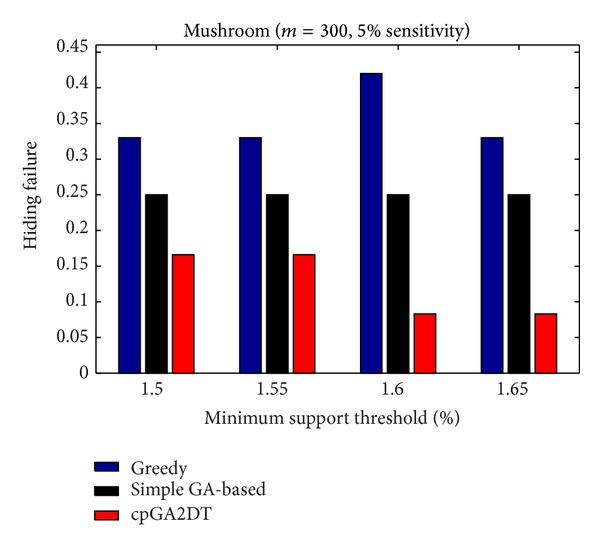
Comparisons of hiding failure at various minimum support thresholds for mushroom database.

**Figure 17:**
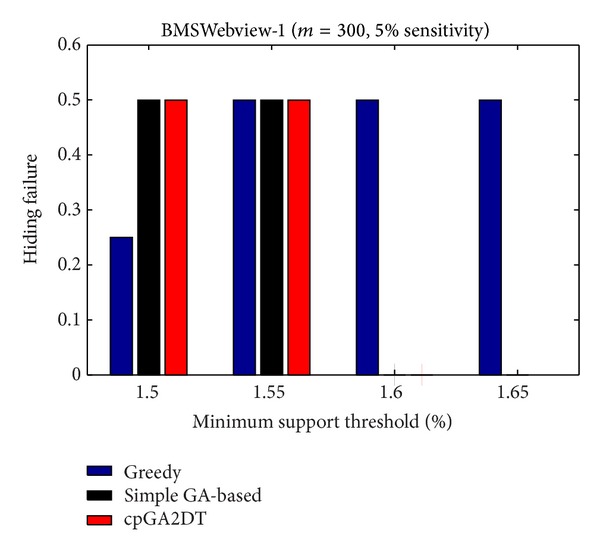
Comparisons of hiding failure at various minimum support thresholds for BMSWebview-1 database.

**Figure 18:**
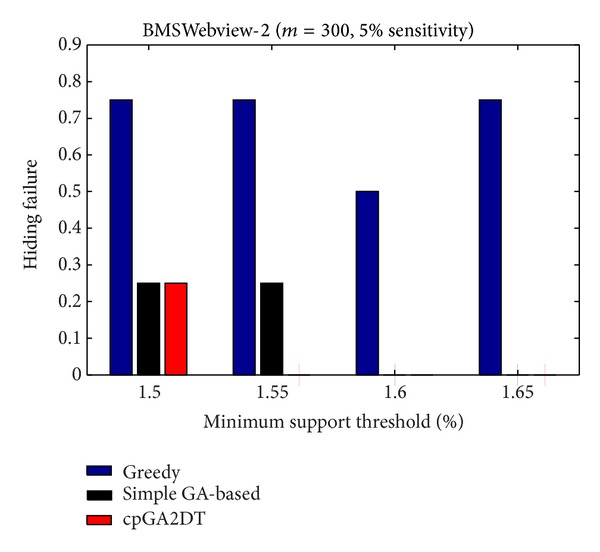
Comparisons of hiding failure at various minimum support thresholds for BMSWebview-2 database.

From Figures 13 to 15, it is obvious to see that the greedy approach has the worst performance for hiding the sensitive itemsets in three databases. The proposed cpGA2DT generally has the best performance for hiding the sensitive itemsets in three databases except when the sensitive percentage is set at 10% of frequent itemsets in BMSWebview-2 database. Experiments are then conducted to show that the performance of hiding failure obtained three algorithms at various minimum support thresholds. The results are then shown in Figures 16, 17, and 18.

From Figures 16 to 18, it is easily found that the proposed cpGA2DT generally has the best performance of hiding failure at various minimum support thresholds for three databases and is better than the greedy and the simple GA-based algorithms in most cases at various minimum support thresholds for three databases.

### 6.3. Missing Cost (MC)

**Figure 19:**
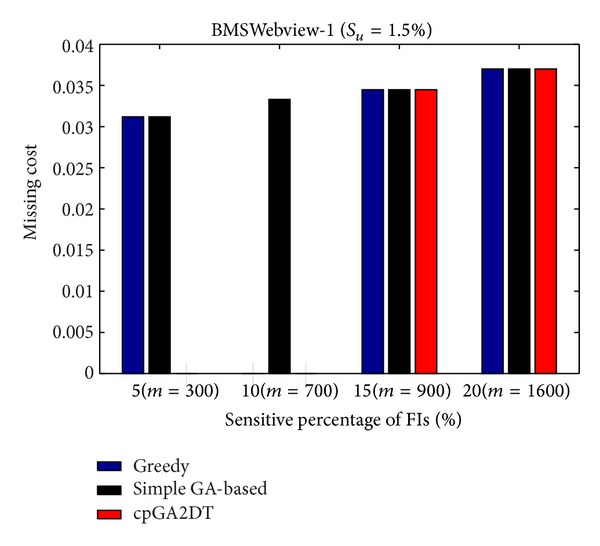
Comparisons of missing cost at various sensitivity percentages of frequent itemsets for BMSWebview-1 database.

**Figure 20:**
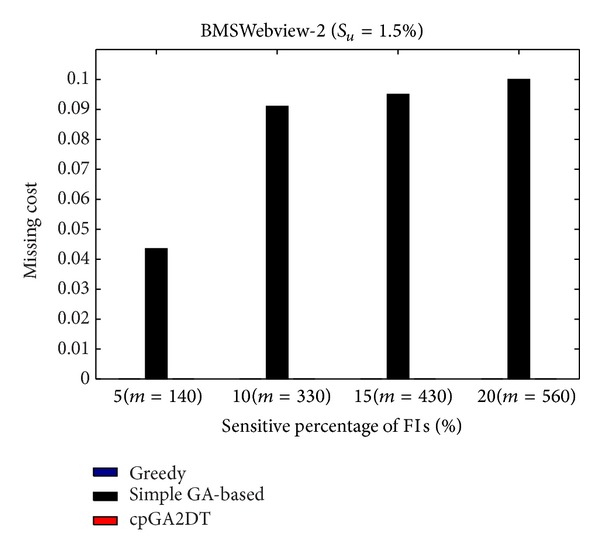
Comparisons of missing cost at various sensitivity percentages of frequent itemsets for BMSWebview-2 database.

The side effects of missing cost are also evaluated to show the performance of the proposed cpGA2DT, which is calculated as

(4) $$\begin{array}{c} \text{MC}=\frac{\left|\text{FIs}\left(D\right)\right|-\left|\text{FIs}\left(D^{*}\right)\right|}{\left|\text{FIs}\left(D\right)\right|}, \end{array}$$

where |FIs(*D*)| is the number of frequent itemsets before data sanitization and |FIs(D*)| is the number of frequent itemsets after data sanitization. Note that even sensitive itemsets are the frequent itemsets but not considered here to calculate the missing cost. The missing cost obtained three algorithms which are then compared at various sensitivity percentages of the sensitive itemsets for three databases with *S*_*u*_ (= 1.5%). The missing cost that obtained three algorithms has, however, zero for the mushroom database since the mushroom database is too small for data sanitization. All sensitive itemsets can thus be successfully hidden without any missing cost in mushroom database. The results for the other two databases are then shown in Figures 19 to 20.

**Figure 21:**
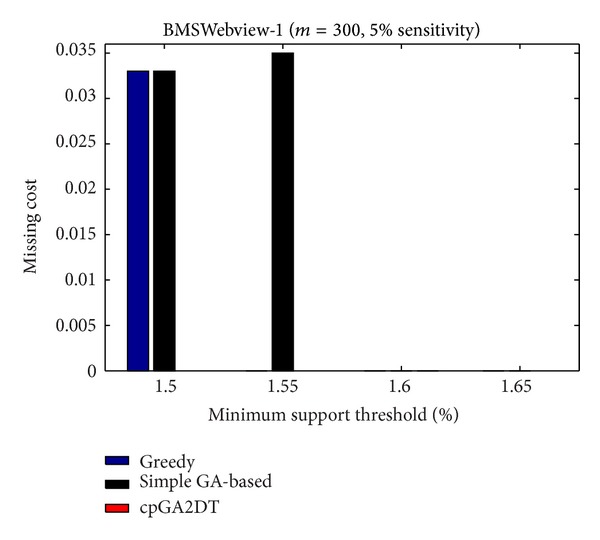
Comparisons of missing cost at various minimum support thresholds for BMSWebview-1 database.

**Figure 22:**
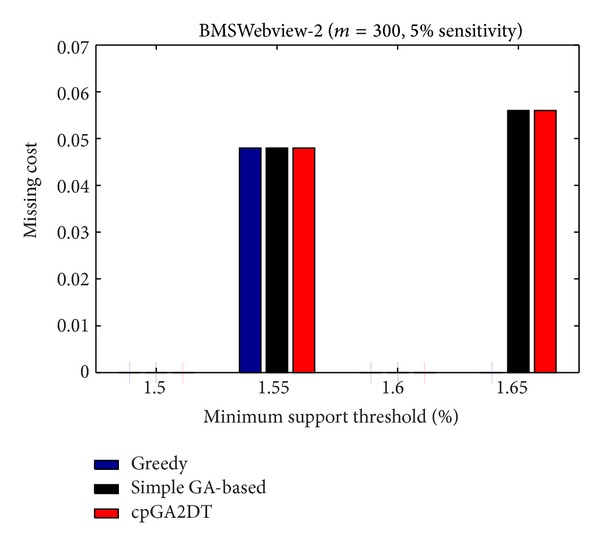
Comparisons of missing cost at various minimum support thresholds for BMSWebview-2 database.

In the experiments of the proposed cpGA2DT, the weight of hiding failure is set at 0.5, which is higher than the missing cost and artificial cost. From Figure 19, the proposed cpGA2DT has generated some missing costs at 15% and 20% sensitive percentages of frequent itemsets. The proposed cpGA2DT has not any missing cost in BMSWebview-2 database. Experiments are then conducted to show that the performance of missing cost obtained three algorithms at various minimum support thresholds for three databases. Again, the missing cost is zero for the obtained three algorithms for mushroom database. The results for the other two databases are then shown in Figures 21 to 22.

From Figure 21, the proposed cpGA2DT algorithm has no missing cost for the BMSWebview-1 database. The greedy approach slightly outperforms better than the proposed cpGA2DT in the BMSWebview-2 but the proposed cpGA2DT still achieves good performance at the 1.5% and 1.6% minimum support thresholds with zero missing cost. In the experimental process, we have also found that the greedy approach is executed to delete transactions from top transactions to down ones, and the deleted transactions of the greedy approach in BMSWebview-2 have fewer numbers of items within it. Thus, the missing cost of the greedy approach is a little bit better than the proposed algorithm at 1.65% minimum support threshold.

### 6.4. Artificial Cost (AC)

The side effects of artificial cost are also evaluated to show the performance of the proposed cpGA2DT, which is calculated as

(5) $$\begin{array}{c} \mathrm{AC}=\frac{\left|\text{FIs}\left(D^{*}\right)\right|-\left|\text{FIs}\left(D^{*}\right)\cap \text{FIs}\left(D\right)\right|}{\left|\text{FIs}\left(D^{*}\right)\right|}. \end{array}$$

In three databases that obtained three algorithms in various sensitivity percentages of the frequent itemsets and various minimum support thresholds, there are not any side effects of artificial cost. For the greedy approach in the experiments, the deleted transactions have short length with lower support items; thus the artificial cost is not shown. For the proposed cpGA2DT, instead of the above reason of the greedy approach, the artificial cost is also considered as a factor in the evaluation process, thus avoiding the side effects of artificial cost.

## 7. Conclusion

In this paper, a compact GA-based cpGA2DT algorithm is thus proposed to hide the sensitive itemsets through transaction deletion. A flexible fitness function with three adjustable weights is also designed to consider the general side effects of hiding failure, missing cost, and the artificial cost to determine the goodness of the chromosomes. The prelarge concept is adopted in the proposed algorithm to reduce the computations of database rescan. The size of the populations is also reduced by the compact GA approach, thus reducing the memory lack problems of traditional GAs. Experiments are conducted to show that the proposed cpGA2DT algorithm outperforms better than the greedy and simple GA-based algorithms considering all criteria of side effects but the execution time.

## Figures and Tables

**Algorithm 1:**
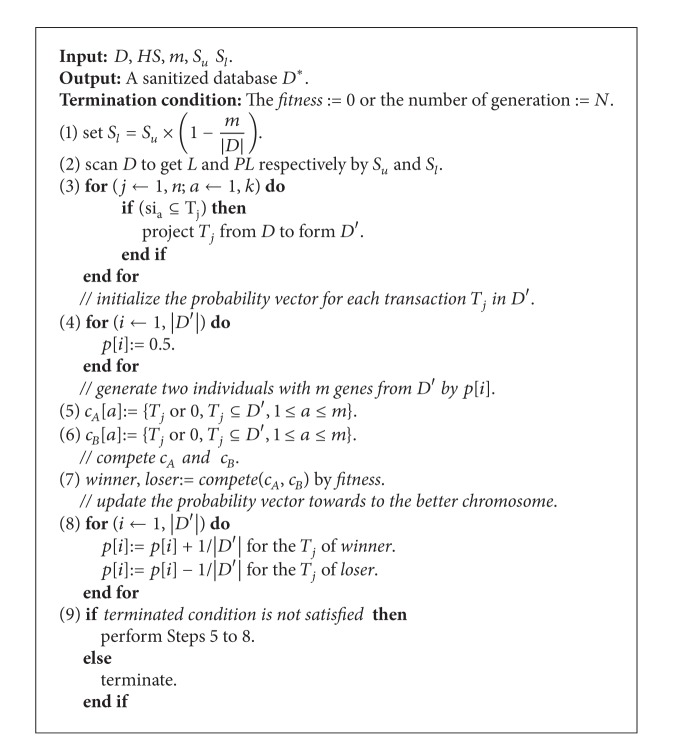
cpGA2DT algorithm.

**Table 1:** Original database.

TID	Item
1	*a*, *b*, *c *
2	*b*, *c*, *e *
3	*a*, *b*, *c*, *e *
4	*a*, *b*, *e *
5	*a*, *b*, *e *
6	*a*, *c*, *d *
7	*b*, *c*, *d*, *e *
8	*b*, *c*, *e *
9	*c *
10	*a*, *b *

**Table 2:** Large itemsets.

1-itemset	Count	2-itemset	Count	3-itemset	Count
*a *	6	*ab *	5	*bce *	4
*b *	8	*bc *	5		
*c *	7	*be *	6		
*e *	6	*ce *	4		

**Table 3:** Prelarge itemsets.

Prelarge 1-itemset	Count	Prelarge 2-itemset	Count	Prelarge 3-itemset	Count
*d *	2	*ac *	3	*abc *	2
		*ae *	3	*abe *	3
		*cd *	2		

**Table 4:** Two individuals.

*C*_*A*_	2	7	8	5
*C*_*B*_	3	2	4	7

**Table 5:** Probability vector.

TID	2	3	4	5	7	8
Probability	0.5	0.667	0.667	0.33	0.33	0.5

**Table 6:** Three databases.

Database	Transactions	Items	Avg. of transactions
Mushroom	8,124	119	23
BMSWebview-1	59,602	497	2.5
BMSWebview-2	77,512	3,340	5

## Notations

*D*: Original database to be sanitized
|*D*|: Number of transactions in *D*
*D*′: Projected database from *D* in which each transaction in *D*′ contains any sensitive itemsets si_*a*_ in HS
*D**: Sanitized database after the designed algorithm
HS: A set of sensitive itemsets to be hidden, HS = {si_1_, si_2_,…, si_*k*_}
*m*: Number of transactions to be deleted for hiding sensitive itemsets
*S*_*u*_: Upper support threshold
*S*_*l*_: Lower support threshold, *S*_*u*_ > *S*_*l*_
*L*: A set of large itemsets in which the count of each itemset is larger than or equal to |*D* | ×*S*_*u*_
PL: A set of prelarge itemsets in which the count of each itemset lies between |*D* | ×*S*_*u*_ and |*D* | ×*S*_*u*_
*p*: Probability vector of transactions in *D*′
*c*_*A*_, *c*_*B*_: Two competition chromosomes.
